# Early real-life evaluation of the efficacy of esketamine in resistant depressive disorder

**DOI:** 10.1192/j.eurpsy.2022.1417

**Published:** 2022-09-01

**Authors:** S. Faisant, P. Crublet, R. Bellay, B. Langrée, N. Marie, M. Rojo-Bouton

**Affiliations:** Centre Hospitalier Guillaume Regnier, Pharmacie, 108 Avenue Du General Leclerc, Rennes, France

**Keywords:** esketamine, therapeutic strategy, resistant depressive disorder, antidepressant

## Abstract

**Introduction:**

The efficacy and of current antidepressants is insufficient. Esketamine, a new antidepressant administered by nasal route, is available since 2019 in the management of resistant characterized depressive episodes.

**Objectives:**

To evaluate the response profile of patients to Esketamine in our institution specialized in mental health.

**Methods:**

We included all patients treated with Esketamine in our institution from November 2019 to September 2021.We collected efficacy and tolerability data using the computerized and paper patient record, prescribing support software, and nursing staff.

**Results:**

Since 2019, we treated 11 patients with Esketamine in combination with an antidepressant as indicated in the MA. Two patients from the 11 were found resistant, three discontinued due to adverse events, four relapsed after an initial clinical response, and two were still ongoing at the end of the study.

**Conclusions:**

Despite an initial and rapid response, our study does not highlight any long-term efficacy of Esketamine in resistant depressive disorder. This highlight the fact that its use in the acute phase of depression or earlier in the management strategy could be a good alternative because of its rapid onset of action. Esketamine was initiated as a last line therapy, which may represent a bias in the evaluation of the molecule, as the later the depression is treated, the lower the response rate. The place of Esketamine in the therapeutic strategy is not yet well determined due to a lack of hindsight, and the question of pharmacological tolerance and dependence on the molecule arises.

**Disclosure:**

No significant relationships.

